# Burkholderia pseudomallei-loaded cells act as a Trojan horse to invade the brain during endotoxemia

**DOI:** 10.1038/s41598-018-31778-8

**Published:** 2018-09-11

**Authors:** Pei-Tan Hsueh, Hsi-Hsun Lin, Chiu-Lin Liu, Wei-Fen Ni, Ya-Lei Chen, Yao-Shen Chen

**Affiliations:** 1 Department of Internal Medicine, Kaohsiung Veterans General Hospital, Kaohsiung, Taiwan; 2 General Clinical Research Center, Medical Research Department, Taipei Veterans General Hospital, Taipei, Taiwan; 3 Institute of Public Health, School of Medicine, National Yang-Ming University, Taipei, Taiwan; 4 Department of Biotechnology, National Kaohsiung Normal University, Kaohsiung, Taiwan; 5 Department of Internal Medicine, National Yang-Ming University, Taipei, Taiwan

## Abstract

Neurologic melioidosis occurs in both human and animals; however, the mechanism by which the pathogen *Burkholderia pseudomallei* invades the central nervous system (CNS) remains unclear. *B. pseudomallei*-loaded Ly6C cells have been suggested as a putative portal; however, during melioidosis, lipopolysaccharide (LPS) can drive disruption of the blood-brain barrier (BBB). This study aims to test whether the Trojan horse-like mechanism occurs during endotoxemia. The expression levels of cerebral cytokines, chemokines and cell adhesion molecules; the activation of astrocytes, microglia and endothelial cells; and the increased vascular permeability and brain-infiltrating leukocytes were evaluated using *B. pseudomallei*, *B. thailandensis*, *B. cenocepacia* and *B. multivorans* LPS-induced brains. Accordingly, different degrees of BBB damage in those brains with endotoxemia were established. The *B. multivorans* LPS-induced brain exhibited the highest levels of disruptive BBB according to the above mediators/indicators. Into these distinct groups of endotoxemic mice, *B. pseudomallei*-loaded Ly6C cells or free *B. pseudomallei* were adoptively transferred at equal bacterial concentrations (10^3^ CFU). The bacterial load and number of cases of meningeal neutrophil infiltration in the brains of animals treated with *B. pseudomallei-*loaded Ly6C cells were higher than those in brains induced by free *B. pseudomallei* in any of the endotoxemic groups. In particular, these results were reproducible in *B. multivorans* LPS-induced brains. We suggest that *B. pseudomallei-*loaded cells can act as a Trojan horse and are more effective than free *B. pseudomallei* in invading the CNS under septic or endotoxemic conditions even when there is a high degree of BBB disruption.

## Introduction

Melioidosis is a fatally infectious disease caused by *Burkholderia pseudomallei* infection that usually occurs in endemic areas, such as northern Australia and Southeast Asia. The clinical spectrum of melioidosis varies and can present as localized cutaneous suppuration, multiple organ abscesses and neurological disorders^1,2^. The severity of melioidosis depends on the invasive route, the size of inoculation, the virulent characteristics of the bacterium and host defense factors^1,3–5^. Splenic and hepatic abscesses that develop secondary to primary foci *via* hematogenous spread are commonly observed, but *B. pseudomallei* can also be isolated from suppurated bone, muscles, or the parotid and prostate glands^6,7^. Patients rarely manifest neurologic melioidosis, which rapidly leads to death when it does occur^6^. While meningoencephalitis is the most common presentation in patients with neurologic melioidosis^2^; the mechanisms by which *B. pseudomallei* invades the central nervous system (CNS) remain unclear.

When inhaled, *B. pseudomallei* can directly invade the CNS by following extensions of the olfactory and trigeminal nerves^8,9^. When an adoptive transfer process that mimicked a Trojan horse was used to model a hematogenous route, circulating *B. pseudomallei-*loaded CD11b^+^Ly6C^+^ monocytes crossed cerebral endothelial cells and induced neurologic melioidosis^10,11^. However, *B. pseudomallei* is an endotoxemia- and sepsis-causing bacteria^12^. During sepsis or endotoxemia, lipopolysaccharide (LPS) triggers cerebrovascular endothelial or glial cells to generate prostanoids, NO and cytokines (TNF-α, IL-1 and IL-6), which can damage tight junctions or degrade the glycocalyx of the blood-brain barrier (BBB) and glial cells when over-expressed^13^. In a murine model of melioidosis, the bacterial load successively appeared in organs from the spleen and liver to the bone marrow before ultimately reaching the brain through hematogenous infection^10^. Once septic melioidosis is established, the CD11b^+^CD115^+^ phagocytic progenitors, CD11b^+^CD14^+^ activated macrophages, CD11b^+^CX_3_CR1^+^ resident cells as well as CD11b^+^CD62L^+^, CD11b^+^CCR2^+^ and CD11b^+^CD31^+^ inflamed cells harbored numerous *B. pseudomallei* in the bone marrow^11^. At this time, a large amount of free *B. pseudomallei* was present in the blood, and the bacteria also existed in a variety of circulating CD11b leukocytes, including Ly6C monocytes, Ly6G neutrophils, F4/80 macrophages and CD19 B cells^10^. Ly6C monocytes were the predominate type of *B. pseudomallei-*loaded cells^10,11^. If the BBB is impaired to a critical degree by endotoxemia in melioidosis, the major mechanisms by which the bacteria colonize the brain are hematogenous infection or *via B. pseudomallei*-loaded cells, which originate directly from circulating cells or indirectly from the bone marrow, that act as a Trojan horse to enter the brain^11^.

Previously, we reported that *B. pseudomallei* vgh07, *B. thailandensis* E264, *B. cenocepacia* BC2 and *B. multivorans* NKI379 LPS induced different degrees of splenic cell activation and bone marrow cell proliferation as well as serum cytokine (TNF-α, IL-1β, IL-6 and IL-17) and chemokine (MCP-1, MIP-1α and RANTES) expression^14^. *B. multivorans* LPS was a potentially stronger stimulant that induced serum inflammatory cytokine production, but *B. thailandensis* LPS was a weaker inducer of the expression of some factors than were other types of *Burkholderia* LPS^14^. Thus, we suspected that there was distinct damage to BBB permeability and/or that endothelial activation occurred in the endotoxemic brains. We hypothesized that if the Trojan horse mechanism was the predominant triggering factor, the number of cases in which neurologic melioidosis was induced by *B. pseudomallei-*loaded Ly6C monocytes would be higher than the number induced by free bacteria in mice with distinct degrees of endotoxemia-induced BBB structural disruption.

## Results

### Up-regulation of cerebral cytokines, chemokines and CAMs

**Figure 1:**
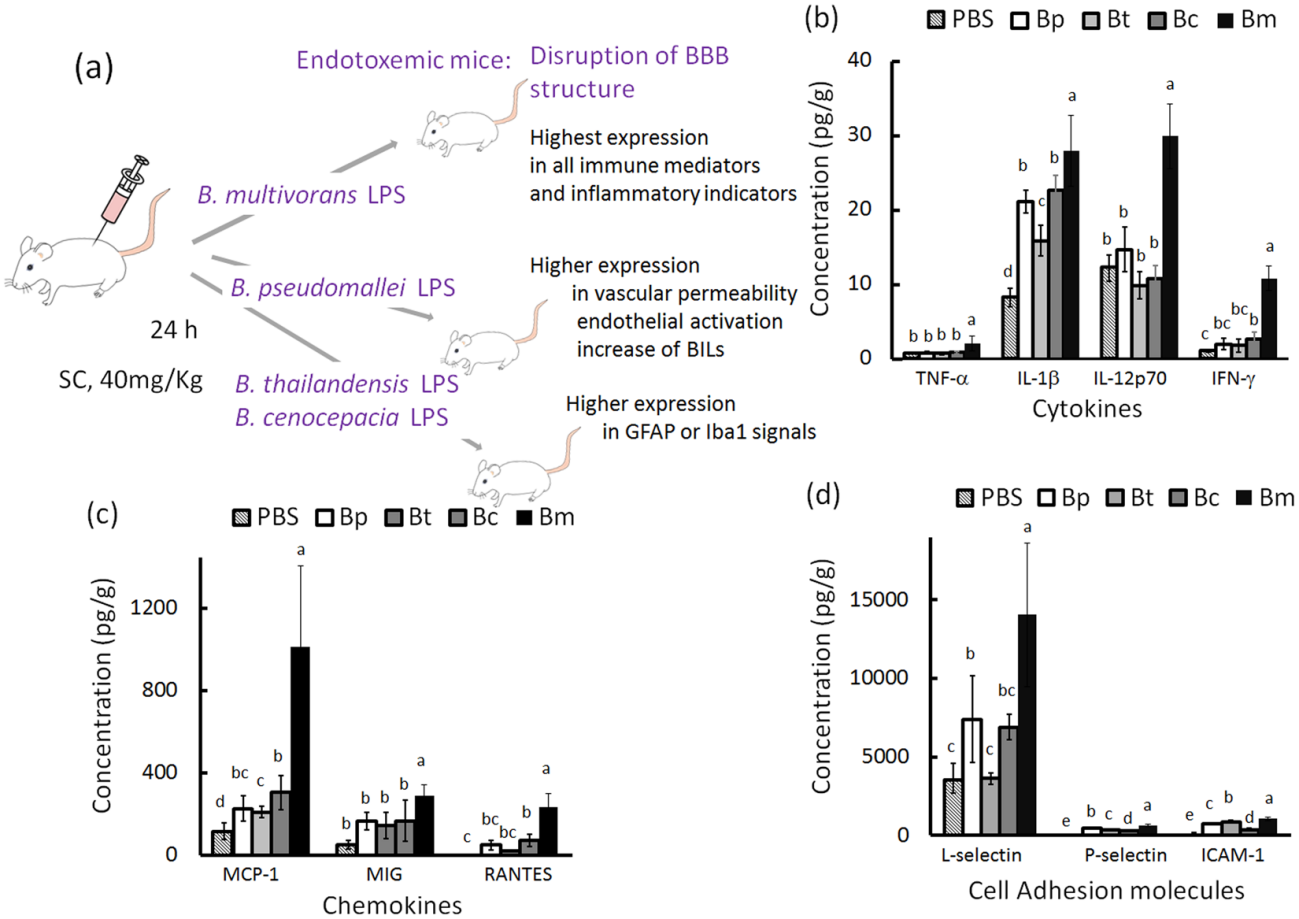
Cerebral cytokines, chemokines, and cell adhesion molecules. The experimental design for endotoxemic mice is shown (**a**). The expression levels of cerebral (**b**) cytokines, (**c**) chemokines and (**d**) CAMs were detected in *Burkholderia* LPS-induced endotoxemic mice (n = 6 per group). The letters a-e represent significant differences at p < 0.05 (one-way ANOVA; Tukey’s HSD test).

Previous studies have shown that systemic cytokines, chemokines and cell adhesion molecules (CAMs) are robustly present in the blood in *Burkholderia* LPS-induced endotoxemic mice^14^. In this study, the levels of cytokines, chemokines, and CAMs were evaluated in the brain tissues in endotoxemic mice (the experimental design is shown in Fig. 1a and the time course data are shown in Table S1). *B. multivorans* LPS was the strongest immunostimulant that induced a high level of expression of cytokines TNF-α (2.2 ± 1.0 pg/g), IL-1β (28.0 ± 4.8 pg/g), IL-12p70 (29.9 ± 4.3 pg/g), and IFN-γ (10.9 ± 1.6 pg/g) (Fig. 1b); the chemokines MCP-1 (1,012 ± 394 pg/g), MIG (289 ± 57 pg/g) and RANTES (237 ± 64 pg/g) (Fig. 1c); and the CAMs L-selectin (14,044 ± 4,567 pg/g), P-selectin (634 ± 94 pg/g) and ICAM-1 (1,066 ± 114 pg/g) on 24 h post-stimulation (Fig. 1d). These concentrations were higher than those observed in the other *Burkholderia* LPS-induced brains or PBS (phosphate buffered saline)-treated controls (F4,25 > 9.69; p < 0.05, Tukey’s HSD test). There were no differences in the expression of the cytokines IL-1α and IL-17a, the chemokines MIP-1α and MIP-1β, or the CAM E-selectin among the evaluated groups of *Burkholderia* LPS-induced brains (Table S1). With the exception of the expression in *B. multivorans* LPS-induced brains, the levels of TNF-α, IL-12p70, IFN-γ, MIG, RANTES and L-selectin were at approximately background levels based on one of the other *Burkholderia* LPS-induced brains. However, the inflammatory response was indeed induced because the levels of IL-1β, MCP-1, P-selectin and ICAM-1 in *B. pseudomallei*, *B, thailandensis* and *B. cenocepacia* LPS-induced brains were significantly increased compared with those in PBS-treated brains (p < 0.05, Tukey’s HSD test). The up-regulation of IL-1β, a predominated cytokine that is involved in resultant BBB leakage^15^, was ranked as follows: *B. multivorans* LPS- > *B. pseudomallei* LPS- = *B. cenocepacia* LPS- > *B. thailandensis* LPS- > PBS-induced brains.

### Characteristics of glial cells in endotoxemic mice

**Figure 2:**
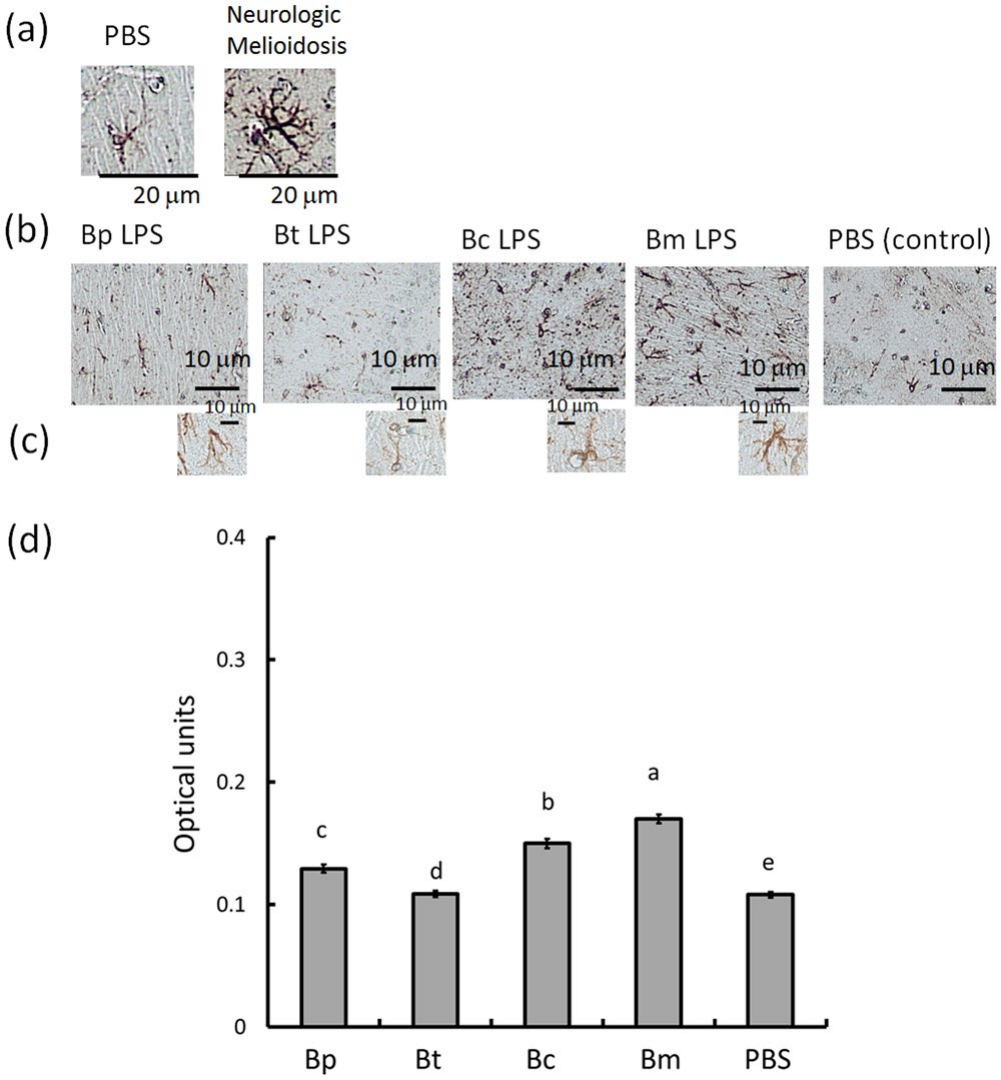
GFAP-expressing astrocytes. Histologic examination was used to identify GFAP-expressing astrocytes in the hippocampus (**a**: left, PBS-treated controls; right, melioidosis brains; both 400X ). *B. pseudomallei* (Bp) LPS-, *B. thailandensis* (Bt) LPS-, *B. cenocepacia* (Bc) LPS- and *B. multivorans* (Bm) LPS-induced brains, and PBS-treated controls are shown ranked from left to right (**b**), all 100X ). A representative GFAP cells in Bp, Bt, Bc, Bm and PBS-induced brain (**c**), from left to right, all 400X ). (**d**) The optical units of the GFAP-positive areas were calculated using ImageJ software (open source, https://imagej.net/Fiji). The letters a-e represent significant differences at p < 0.05 (one-way ANOVA; Tukey’s HSD test).

Reactive astrogliosis (astrocyte proliferation or progressive cellular hypertrophy) is a hallmark of severe CNS infection^16^. For example, the robust soma and branches can be observed during neurologic melioidosis for the BALB/c mice (Fig. 2a: left, a PBS-induced brain; right, a brain with melioidosis). During endotoxemia, the GFAP (glial fibrillary acidic protein, astrocyte markers)-positive cells in the brains were localized at perivascular cuffs (Fig. 2b,c; in the hippocampus), but astrocyte proliferation and glial scar formation were not observed. The intensity of the GFAP signals was significantly increased following *B. multivorans* LPS- (0.170 ± 0.004 units) > *B. cenocepacia* LPS- (0.150 ± 0.004 units) > *B. pseudomallei* LPS- (0.129 ± 0.003 units) > *B. thailandensis* LPS- (0.109 ± 0.002) > PBS-induced brains (0.107 ± 0.002 units) (Fig. 2d; F4,25 = 400.75; p < 0.05, Tukey’s HSD test).

**Figure 3:**
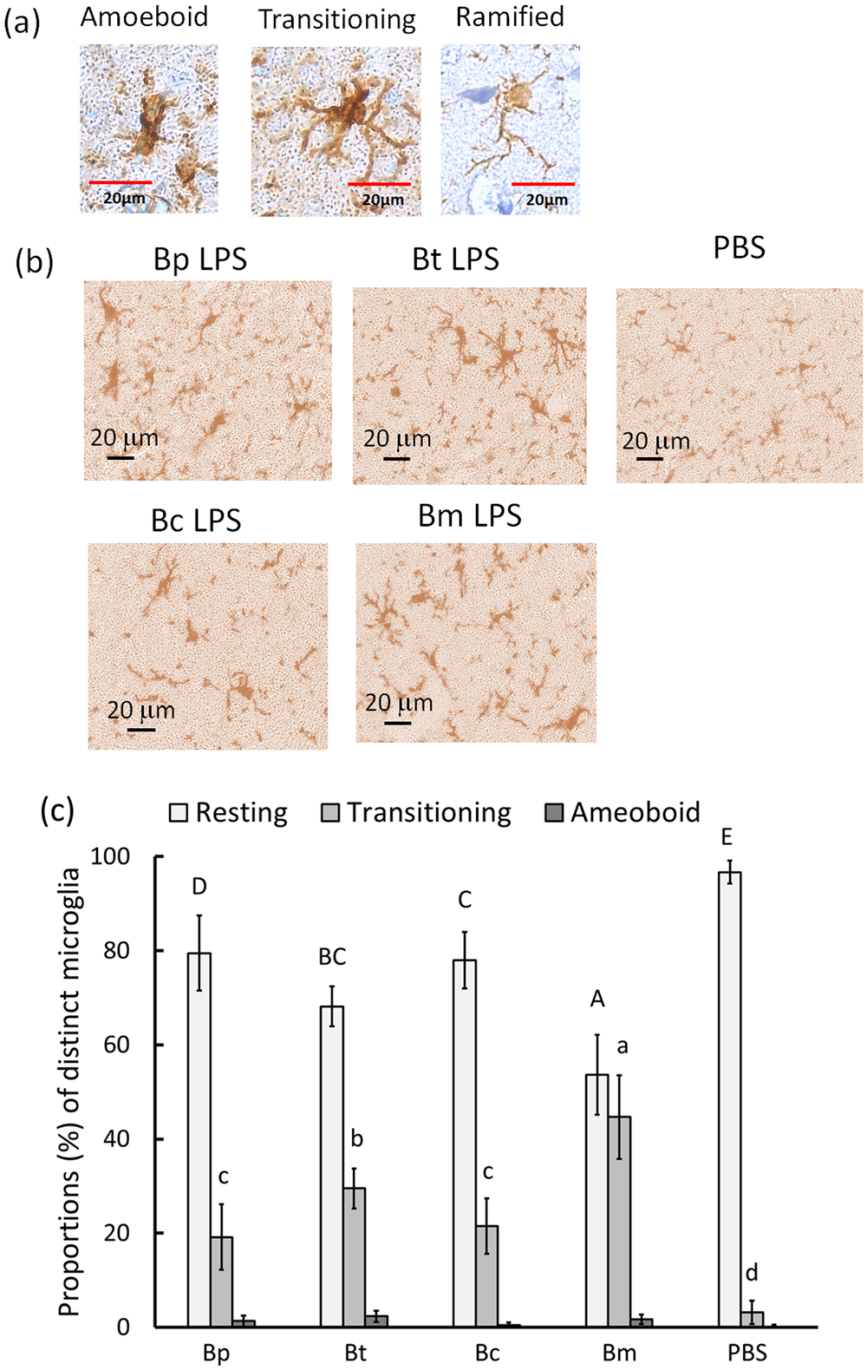
Iba1-expressing microglia. The microglia were categorized as amoeboid, transitioning or ramified, as shown (**a**; 400X ). The Iba1-expressing microglia in the cerebral cortex in four kinds of Burkholder LPS-induced and PBS-treated brains are shown (**b**; 400X ; processed by ImageJ software [H DBA mode]). The proportions of each microglia category out of 100 microglia are shown (**c**). The letters a-d or A-E represent significant differences at p < 0.05 (one-way ANOVA; Tukey’s HSD test).

Phenotypically, resting ramified microglia were found in PBS-treated brains. However, activated amoeboid (round soma, shrunk branches) or transitioning (enlarged soma, thick branches) microglia were present in melioidosis brains (Fig. 3a, where in the representatives of three types of microglia in the cerebral cortex are shown). The Iba (ionized calcium binding adaptor molecule)-1-expressing microglia were observed in *Burkholderia* LPS-induced brains (Fig. 3b), and the percentages of the ramified, transitioning and amoeboid forms of microglia out of 100 microglia were calculated. In *B. multivorans* LPS-induced brains, 54 ± 6% of the microglia were resting ramified, whereas >68 ± 4% of the microglia were found in other *Burkholderia* LPS-induced brains (Fig. 3c; F4,25 = 38.47; p < 0.05, Tukey’s HSD test). Significant differences at p < 0.05 (Tukey’s HSD test) occurred in the proportion of activated transitioning microglia in the following order: *B. multivorans* LPS- (44.7 ± 8.9%) > *B. thailandensis* LPS- (29.5 ± 4.2%) > *B. cenocepcia* LPS- (21.5 ± 5.9%) = *B. pseudomallei* LPS- (19.2 ± 7.0%) > PBS-induced (3.2 ± 2.5%) brains. Amoeboid (activated) microglia were rarely found in any of the endotoxemic brains (<0.5% for all endotoxemia models).

### Endothelial activation, vascular permeability and brain-infiltrating leukocytes

**Figure 4:**
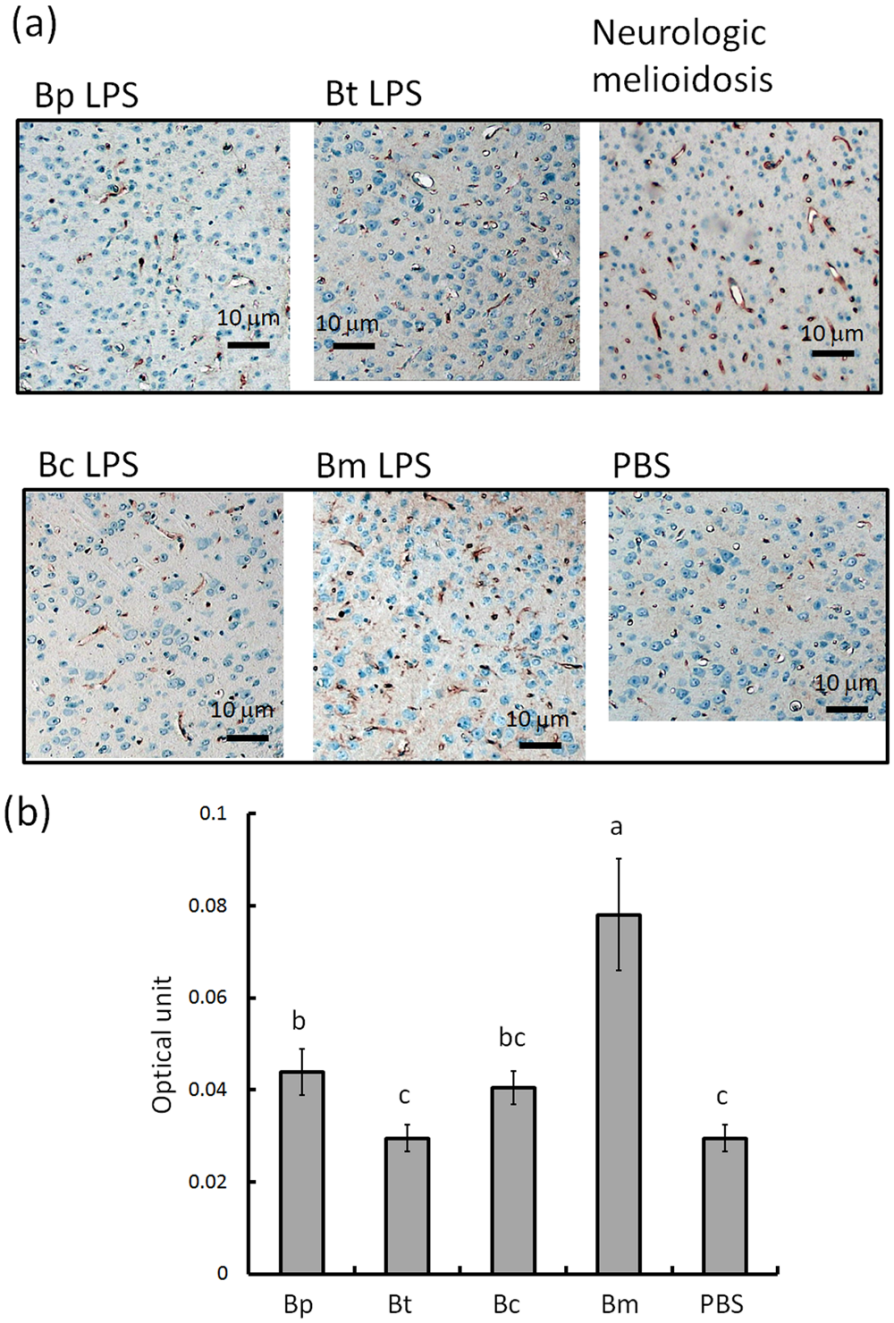
Endothelial activation. The expression of the endothelial marker CD34 in the cerebral cortex is shown in representatives of four types of *Burkholderia* LPS-induced, PBS-treated and neurologic melioidosis brains (**a**). The optical units of the CD34-positive areas were calculated using ImageJ software (open source, https://imagej.net/Fiji) (**b**). The letters a-c represent significant differences at p < 0.05 (one-way ANOVA; Tukey’s post hoc test).

CD34-expressing cells are involved in the activation of the endothelial structures that allow leukocytes to transmigrate into the brain^17^. With the exception of *B. cenocepacia LPS- and B. thailandensis* LPS-induced brains, we found that CD34 levels were increased in brains with endotoxemia (Fig. 4a). There was 0.044 ± 0.005, 0.030 ± 0.003, 0.040 ± 0.004, 0.078 ± 0.012 and 0.029 ± 0.003 units of CD34 signals in *B. pseudomallei*, *B. thailandensis*, *B. cenocepacia*, and *B. multivorans* LPS-induced and PBS-induced brains, respectively (Fig. 4b). *B. multivorans* LPS induced a higher level of CD34 in endothelial cells than that observed in other *Burkholderia* LPS-induced brains (F4,29 = 54.55; p < 0.05, Tukey’s HSD test).

**Figure 5:**
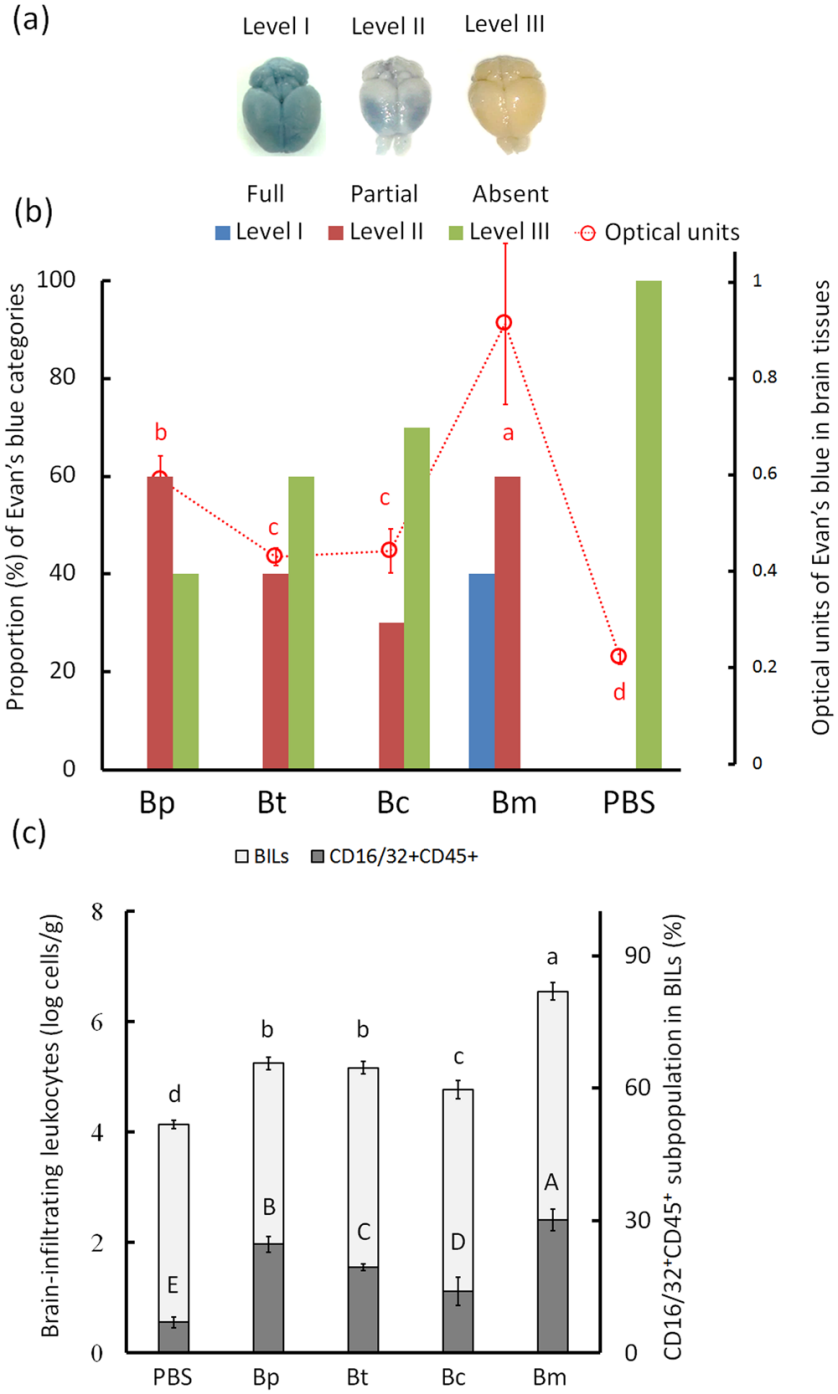
Vascular permeability and brain-infiltrating leukocytes. The endotoxemic mice were intravenously injected with Evan’s blue dye (2% in saline, 4 mL/kg). After one hour, the distribution of Evan’s blue was used to categorize the brains into three groups: level I (full distribution), level II (partial distribution and level III (absent) (**a**). The proportions (%) of brains that were level I versus level III in each *Burkholderia* LPS-induced group as well as the optical units of Evan’s blue in the homogenized brains are shown (**b**). Brain-infiltrating leukocytes (BILs) were collected from endotoxemic mice. The total number of BILs and the proportion of CD16/32^+^CD45^+^ cells are shown (**c**). The letters a-d or A-E represent significant differences at p < 0.05 (one-way ANOVA; Tukey’s HSD test).

Evan’s blue (an azo dye) has a high affinity for serum albumin and cannot penetrate across the BBB^18^. The distributions of hematogenous Evan’s blue in the organs (the liver, heart, lung, spleen and brain) of animals with endotoxemia are described in Table S2. The dye-infiltrated brain was categorized into three levels, as follows: level I, fully distributed; level II, partially distributed; and level III, absent (Fig. 5a). Approximately 40% and 60% of the *B. multivorans* LPS (the strongest stimulant)-induced brains were level I and level II, respectively. In the *B. pseudomallei* LPS-induced endotoxemic brains, none were level I, and 60% were level II. Approximately 60%-70% of the *B. cenocepacia* or *B. thailandensis* LPS-induced brains were level III (Fig. 5b). The average number of optical units in the homogenized dye-infiltrated brain was ranked as follows: *B. multivorans* LPS- (0.914 ± 0.167 units) > *B. pseudomallei* LPS- (0.591 ± 0.049 units) > *B. cenocepacia* LPS- (0.443 ± 0.046 units) = *B. thailandensis* LPS- (0.430 ± 0.017 units) > PBS-induced (0.225±0.016 units) brains (Fig. 5b; F4,25 = 59.86; p < 0.05, Tukey’s HSD test).

To determine whether cerebral leukocyte migration was expanded during endotoxemia, total infiltrating cells were measured in endotoxemic brains. Compared with PBS-induced brains, the level of brain-infiltrating leukocytes (BILs) was higher in the endotoxemic mice for all strains. *B. multivorans* LPS was the most potent inducer, generating 6.56 ± 0.16 × 10^6^ cells/g, whereas 4.77 ± 0.16 × 10^4^ to 5.25 ± 0.11 × 10^5^ cells/g were generated in other Burkholderia LPS-induced brains, as follows: *B. multivorans* LPS- > *B. pseudomallei* LPS- = *B. thailandensis* LPS- > *B. cenocepacia* LPS- > PBS-induced brains (Fig. 5c, F4,25 = 284.96; p < 0.05, Tukey’s HSD test). The CD16/32^+^CD45^+^ cells represent circulating phagocytes, lymphoid cells, NK cells and B cells or activated microglia^11^. These cells have been reported to be capable of carrying *B. pseudomallei* during neurologic melioidosis^10,11^. Undetectable or very low expression of CD16 or CD45 markers are typically present in resident monocyte/microglia and in some patrolling monocytes or lymphocytes and dendritic cells as well as in non-leukocytic pericyte or astrocytes^11,19^. If the Trojan horse hypothesis is true, the CD16/32^+^CD45^+^ cells potentially provide a vector to accommodate *B. pseudomallei*. With respect to our results, the *B. multivorans* LPS-induced brains contained higher amounts of CD16/32^+^CD45^+^ cells (30.1 ± 2.4%) than were observed in other *Burkholderia* LPS-induced brains (14.08 ± 3.2% to 24.8 ± 1.8%; F4,25 = 112.81; p < 0.05, Tukey’s HSD test) (Fig. 5c). The ability to induce infiltration of CD16/32^+^CD45^+^ cells was ranked as follows: *B. multivorans* LPS- > *B. pseudomallei* LPS- > *B. thailandensis* LPS- > *B. cenocepacia* LPS- > PBS-induced brains (p < 0.05, Tukey’s HSD test).

### Neurologic melioidosis

**Figure 6:**
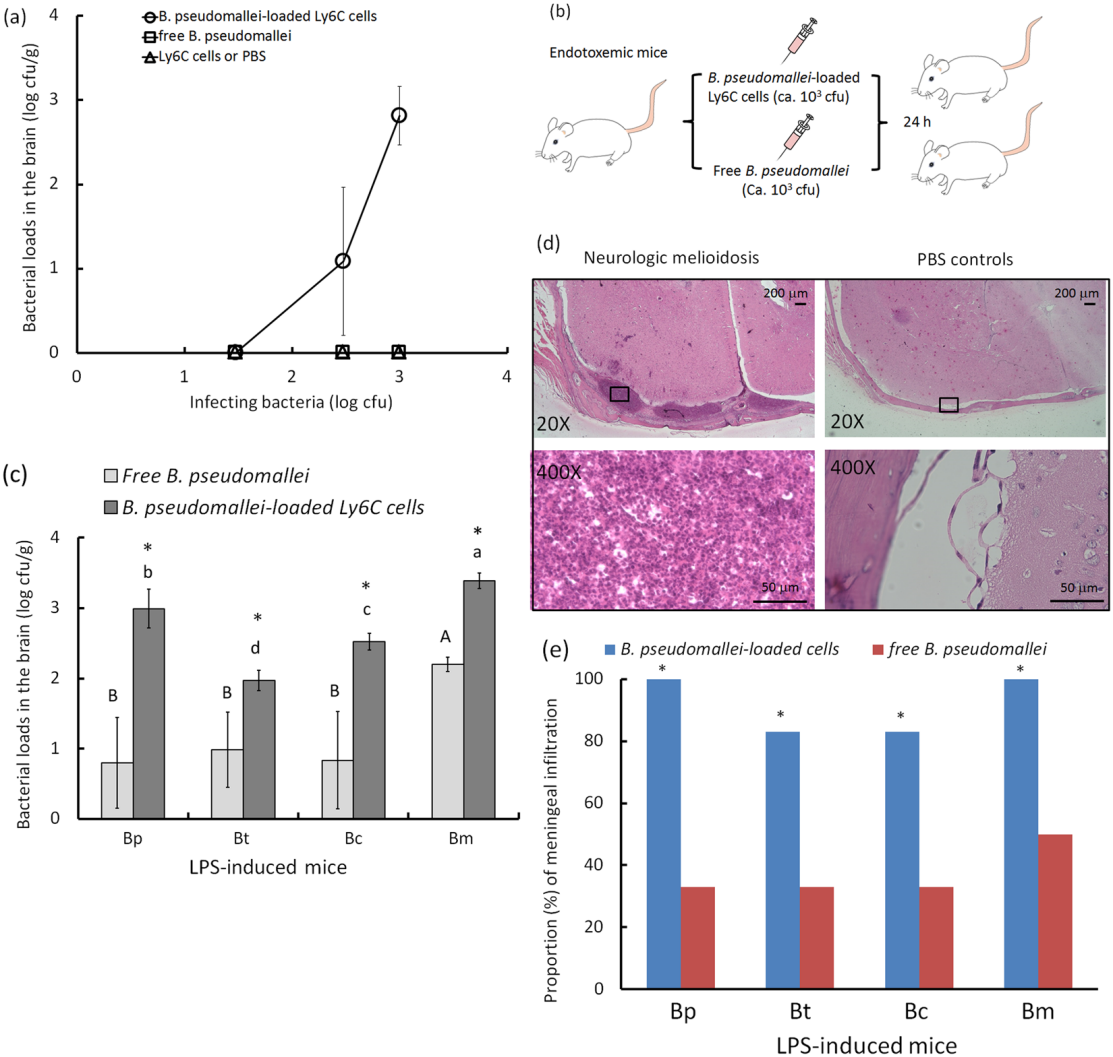
Bacterial loads in the brain and meningeal neutrophil infiltration. The dose-response curves for bacterial loads in the brain (CFU/g) were determined for the healthy mice that were adoptively transferred with *B. pseudomallei*-loaded Ly6C cells (2.5 × 10^2^ cells, 30 CFU; 3.6 × 10^3^ cells, 300 CFU; 4.2 × 10^4^ cells, 1000 CFU) and free *B. pseudomallei* (30–1000 CFU). PBS or empty Ly6C cells were used as controls (**a**). To detect the bacterial loads in the brain in endotoxemic mice, the experimental design of neurologic melioidosis in endotoxemic mice is shown (**b**). After 24 h, the bacterial loads in the brain of the endotoxemic mice that were adoptively transferred with *B. pseudomallei*-loaded Ly6C cells (4.2 × 10^4^ cells, 10^3^ CFU) and free *B. pseudomallei* (10^3^ CFU) are shown. The letters a-d or A-B represent significant differences at p < 0.05 (one-way ANOVA; Tukey’s HSD test). Asterix represent significant difference at p < 0.05 (t-test; comparison between *B. pseudomallei*-load cell group and free *B. pseudomallei* group in any of four endotoxemic groups) (**c**). After adoptive transfer, the representative tissues with meningeal neutrophil infiltration in the parietal lobes are shown (**d**): meningeal neutrophil infiltration, upper left, 100X and bottom left, 400X ; PBS-treated control, upper right, 100X and bottom right, 400X ). The proportions of brains showing meningeal neutrophil infiltration in the *B. pseudomallei* (Bp) LPS-, *B. thailandensis* (Bt) LPS-, *B. cenocepacia* (Bc) LPS- or *B. multivorans* (Bm) LPS-induced groups are shown (**e**). Asterix represent significant difference at p < 0.05 (Fisher’s exact test, one-tailed; comparison between *B. pseudomallei*-load cell group and free *B. pseudomallei* group in any of four endotoxemic groups).

According to our hypothesis, *B. pseudomallei-*loaded cells should act as a route for the development of neurologic melioidosis in endotoxemic mice. Bone marrow Ly6C monocytes are the predominate cells that, during inflammation, can harbor plentiful *B. pseudomallei* and migrate into the brain from the bone marrow^10,11^. Thus, Ly6C donor cells were isolated from the bone marrow of mice with melioidosis and adoptively transferred into endotoxemic mice. First, a dose-response curve was established at 2 d for the bacterial loads in the brains of the mice adoptively transferred with *B. pseudomallei*-loaded Ly6C cells. No bacterial load was detected in the melioidosis brains if the mice were intravenously infected with free *B. pseudomallei* on day 2 post-infection (Fig. 6a). Next, we determined whether *B. pseudomallei*-loaded cells were more capable than free *B. pseudomallei* of brain infection when the BBB was disrupted by endotoxemia (Fig. 6b, experimental design for endotoxemic mice adoptively transferred with *B. pseudomallei*-loaded Ly6C cells or free *B. pseudomallei*). According previous results, four *Burkholderia* LPS-induced brains reflected distinct BBB dysfunction based on different potentials with respect to the expression of immune mediators (cytokines, chemokines and CAM) and inflammatory indicators (activation of endothelial and glial cells and increased vascular permeability, total BILs and subpopulation CD16/32^+^CD45^+^ cells) in the brain. *B. mulltivorans* LPS was the most potent with respect to BBB damage. Compared with bacterial loads in other *Burkholderia* LPS-induced brains, the bacterial numbers counted from *B. multivorans* LPS-induced brains were significantly increased, using the samples performed either by adoptive transfer with *B. pseudomallei*-loaded cells or free *B. pseudomallei* (Fig. 6c, *B. pseudomallei*-loaded Ly6C cells, F3,20 = 71.716; free *B. pseudomallei*, F3,20 = 8.957; p < 0.05, Tukey’s HSD test). However, in any of four endotoxemic groups that represented distinct degrees of BBB dysfunction, *B. pseudomallei*-loaded cells induced greater bacterial loads in the brain compared with free *B. pseudomallei* (Fig. 6c; p < 0.05, t-test).

Meningeal neutrophil infiltration, a characteristic that is commonly observed in the mice with neurologic melioidosis, was typically present in the parietal lobe (Fig. 6d, upper left, meningeal neutrophil infiltration, 100X ; bottom left bottom, 400X ). No neutrophil infiltration was ever observed in the PBS-treated controls (Fig. 6d, upper right, 100X ; bottom right, 400X ). The proportion (%) of meningeal neutrophil infiltration was measured in four endotoxemic groups adoptively transferred with *B. pseudomallei*-loaded cells or free *B. pseudomallei*. Over 80% meningeal neutrophil infiltration was detected in the endotoxemic mice adoptively transferred with *B. pseudomallei*-loaded cells, but less than 50% was detected in the endotoxemic mice infected with free *B. pseudomallei* in any of four *Burkholderia* LPS-induced brains (Fig. 6e; each group, p < 0.05, one-tailed, Fisher’s exact test).

## Discussion

**Figure 7:**
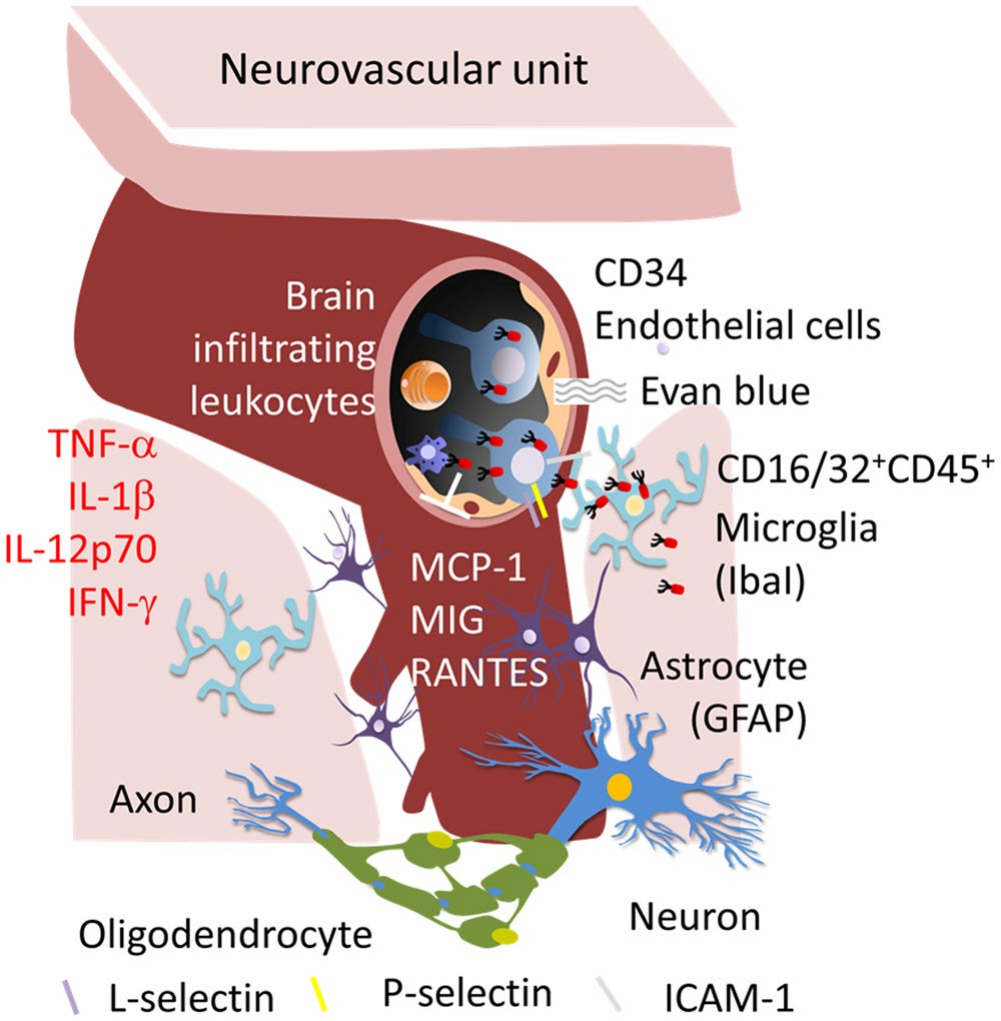
Summary of the Trojan horse mechanism that is activated during endotoxemia. During endotoxemia, the levels of brain cytokines (TNF-α, IL-1β, IL-12p70 and IFN-γ), chemokines (MCP-1, MIG, and RANTES) and CAMs (L-selectin, deep gray; P-selectin, yellow; and ICAM-1, light gray) were increased. GFAP-expressing astrocytes and Iba-1-expressing microglia were activated. In the neurovascular unit, of the number of endothelial CD34 cells, the degree of vascular permeability as measured by Evan’s blue, and brain leukocyte infiltration were increased. Neurologic melioidosis is induced *via B. pseudomallei*-load Ly6C cells (circulating CD16/32^+^CD45^+^ cells) but is not induced *via* free *B. pseudomallei*.

In this study, we first established a model of endotoxemia in which mice exhibited distinctly different degrees of BBB damage. As summarized in Table S3, B. *multivorans* LPS is the strongest stimulant that induced the up-regulation of all immune mediators (items, n = 10) and inflammatory indicators (items, n = 6). Four mediators (IL-1β, MCP-1, P-selectin and L-selectin) and four indicators (endothelial activation, vascular permeability, the numbers of BILs and the proportions of CD16/32^+^CD45^+^ cells) in *B. pseudomallei* LPS-induced brain exhibited the second highest expression. The expression of several mediators, such as TNF-α, IL-12p70, IFN-γ, MIG and L-selectin, as well as endothelial activation in both *B. thailandensis* and *B. cenocepacia* LPS-induced brains was nearly equal to the background of PBS-induced brains. However, the performance of astrocytes (GFAP signaling) or microglia (the numbers of transitioning cells) in *B. cenocepacia* LPS- or *B. thailandensis* LPS-induced brain was more active than the expression in *B. pseudomallei* LPS-induced brains. The fatty acid chain length and the kinds or repeated numbers of sugar moieties in lipid A, in terms of pathogens, leads to different changes in cytokines, chemokines and CAM expression and/or in the BBB measurements such as glial and endothelial activation, vascular permeability and the changes of infiltrating cells^14,20–22^. Septicemia is the most severe form of melioidosis in both human and animals^11,23^. In this study, the bacterial load was higher in endotoxemic brains that were adoptively transferred with *B. pseudomallei*-loads Ly6C cells than in those induced with free *B. pseudomallei* in any of *Burkholderia* LPS-induced brains. This likely represents a Trojan horse mechanism by which hematogenous Ly6C cells harbor *B. pseudomallei* during sepsis or endotoxemia that is commonly seen in melioidosis patients, thereby allowing the bacteria to invade the brain (Fig. 7).

In BALB/c models, neurologic melioidosis was secondary to the presence of primary splenic, hepatic or bone marrow abscesses, from which the cells spread *via* hematogenous routes^10^. The adoptive transfer of *B. pseudomallei*-loaded CD11b cells *via* the tail vein resulted in abscesses in the brain that were predominately localized in the regions surrounding the cerebral superior sagittal sinus^10^. In children with melioidosis who develop meningoencephalitis, brain abscesses are more often localized in the parietal lobe than in other regions^2^. In this study, the endotoxemic mice often exhibited abscesses localized in the dural venous sinus of the parietal lobe. The vascular unit in the parietal lobe likely is a portal for *B. pseudomallei* entry *via* hematogenous spread.

The circulating LPS was detected at 24 h post-administration and peaked after 6 h^13,14^. At 24 h post-administration, LPS induced the robust expression of cytokines, chemokines and CAMs in the blood; however, nearly all tested immune mediators were subsequently down-regulated at 48 h^14^. In this study, the time course of these immune mediators was similar between the blood and the brain, with expression higher at 24 h and then lower at 48 h post-administration. For example, the levels of IFN-γ, MCP-1, RANTES and E-selectin at 48 h were lower than that at 24 h post-stimulation in the cases of *B. multivorans* LPS-induced brain. These findings are in agreement with a previous study that showed that BBB disruption occurred at 24 h following LPS injection^24^. In terms of the time-courses observed in mice intravenously infected with *B. pseudomallei*, in pervious study, the concentration of *B. pseudomallei* declined until these bacteria were undetectable in the blood on day 4 post-infection, but they subsequently re-appeared on day 7 post-infection^10^. During the quiescent period, serum cytokine, chemokine and CAM levels declined; however, TNF-α, IFN-γ and MCP-1 levels then increased on day 7 post-infection in parallel with the appearance of neurological signs in mice with melioidosis^10^. The large amounts of immune mediators that are generated during endotoxemia or sepsis can potentially lead to BBB disruption, which allows *B. pseudomallei-*loaded cells to enter into the brain in the animal model. Indeed, in this study, the degree of BBB functional changes was different in mice induced by different *Burkholderia* LPS, and the colonization of the brain by *B. pseudomallei* was higher following the adoptive transfer of *B. pseudomallei*-loaded Ly6C cells. This was particularly true in *B. multivorans* LPS-induced brains, which showed high levels of the cytokines TNF-α, IL-1β, IL-12p70 and IFN-γ, the chemokines MCP-1, MIG and RANTES, and the CAMs L-selectin, P-selectin and ICAM-1. L-selectin is expressed on leukocytes and belongs to the lectin family of single-chain transmembrane CAMs that bind to sugar moieties on CD34 molecules on endothelial cells^25^. A high level of L-selectin and its complementary molecule CD34 on endothelial cells is suggestive of a putative increase in the recruitment of monocytic Ly6C cells to the brain^26,27^. Moreover, pyrogenic cytokines (TNF-α and IL-1β) can potentially increase the release of MCP-1 from endothelial cells and attract L-selectin-expressing monocytes transmigrating into the brain in addition to causing *B. pseudomallei*-infected cells to pass into the brain *via* selectin- and integrin (ICAM-1)-mediated mechanisms^28^ (Fig. 7).

It is unclear whether effectors, such as LPS, can fully penetrate the BBB. However, low concentrations of an amino moiety of LPS were detected in homogenized brain tissues^13^. In addition, the expression of mRNA of the TLR4 gene, which is responsible for LPS signaling, was detected in brain endothelial cells, microglia and astrocytes^29^. An intact neurovascular unit consists of endothelial cells (CD34), microglia (IbaI), astrocytes (GFAP) and neurons and contributes to the release of major cytokines and chemokines and the recruitment of infiltrating leukocytes. These processes are regulated by interactions among neurons, glia, and immune cells^30^. Immunohistochemistry (IHC) demonstrated that endothelial cells, microglia and astrocytes were activated in *Burkholderia* LPS-induced endotoxemic mice. An increased vulnerability to vascular permeability and augmented numbers of BILs were found in *B. multivorans* LPS-induced brains than in other *Burkholderia* LPS-induced brains, indicating that the most severe form of BBB dysfunction was induced by *B. multivorans* LPS-induced endotoxemia (Fig. 7). In fact, the number of bacteria in the brain was higher in the *B. multivorans* LPS-induced group than in the other *Burkholderia* LPS-induced groups. With the exception of LPS, some somatic antigens, capsular polysaccharide, adhesins, specialized secretion systems, actin-based motility and various secreted factors in *B. pseudomallei* strains as well as multinucleated giant cells formed during infection can together contribute to intracellular survival and bacterial entry to host^31,32^.

The brain is an immunologically privileged organ because the BBB prevents the penetration of bacteria. However, meningitis-causing bacteria, such as *Escherichia coli* K1, *Haemophilus influenzae* and *Neisseria gonorrhoeae*, express specific ligands that allow them to bind to and be internalized by cerebral endothelial cells *via* transcellular or paracellular mechanisms^33^. No specific ligand has been reported for *B. pseudomallei* infection, although *B. mallei*-like variation in the *bimA* motility gene has been reported to be associated with an increased neurotropic threat^34^. Two direct routes of invasion into the brain that bypass the BBB are infection *via* the olfactory and trigeminal nerves *via* inhalation^8,9^. Alternatively, *Listeria monocytogenes* and *Mycobacterium tuberculosis* have been reported to use macrophages as a Trojan horse to transmigrate into the brain *via* leukocyte selectin- and integrin-mediated mechanisms^35,36^. Previously, we demonstrated that *B. pseudomallei-*loaded CD11b leukocytes expressing L-selectin or L-selectin-expressing Ly6C monocytes may also represent Trojan horse-induced mechanism underlying neurologic melioidosis^10,11^. In fact, *B. pseudomallei* is an intracellular pathogen that persists in a variety of cells, such as myeloid progenitors, inflamed monocytes and lymphocytes, NK cells, and resident monocytes/macrophage/lymphocytes^11^. By studying endotoxemia and sepsis, which are commonly observed in melioidosis patients and animal models and are likely to result in BBB disruption, we provide the first demonstration showing that a Trojan horse mechanism exists in septic or endotoxemic melioidosis.

## Materials and Methods

### Strains and Animals

*B. pseudomallei* vgh19 (BioSample: SAMN03372474)^37^, *B. cenocepacia* BC2^38^, *B. multivorans* NKI379 (BioSample: SAMN04008114)^39^ and *B. thailandensis* E264 (ATCC700388) were used in this study. All strains were kept in Luria-Bertani (LB) broth at 37 °C. Manipulations of viable *B. pseudomallei* (Risk group 3) were performed in a Biosafety Level III laboratory at Kaohsiung Veterans General Hospital (Taiwan). BALB/c mice (females, 8–10 weeks of age) were purchased from the National Laboratory Animal Center (NLAC, Taipei, Taiwan). All of the animal experiments in this study were approved by the Institutional Review Board at National Kaohsiung Normal University, Taiwan. Animal care was performed in accordance with the guidelines of the Personal Information Protection Act (Taiwan).

### LPS preparation

LPS extraction was conducted with the hot aqueous-phenol method^14^. The quality of each LPS was ensured based on (1) typical ladder profiles of electrophoretic phenotypes as they appeared in silver staining^20^, (2) the molar ratio of [3-hydroxytetradecanoic acid] to [3-hydroxyhexadecanoic acid] was 1.2–1.4 and was derived from gas chromatography–mass spectrometry (GC-MS) analyses, and (3) over 0.98 of the correlation coefficient (R^2^) derived from the concentration of 3-hydroxytetradecanoic acid (detected by GC-MS) and endotoxin units (detected by a Limulus Amoebocyte Lysate Assay kit, Seikagaku, Tokyo, Japan) (Table S4)^14^. All of the LPS preparations were stored in PBS at −80 °C.

### Experimental design

To establish endotoxemic mice, BALB/c (females, 8–10 weeks of age) mice were subcutaneously injected with 100 μL of *B. pseudomallei*, *B. thailandensis*, *B. cenocepacia* and *B. multivorans* LPS (40 mg/kg). As controls, the animals were injected with equal volumes of PBS. After 24 h or 48 h, the levels of cytokines, chemokines and CAMs were measured in homogenized brain tissues. The number of glial (astrocytes and microglia) and endothelial cells, the vascular permeability and the number of BILs were evaluated (Fig. 1a; protocols, see below).

To test the development of neurologic melioidosis during endotoxemia, the endotoxemic mice (at an 24 h-induction of *Burkholderia* LPS) were intravenously re-injected with 1 × 10^3^ CFU of free *B. pseudomallei* or 4.2 × 10^4^ infected Ly6C cells that harbored exactly 10^3^ CFU of cultivated *B. pseudomallei* vgh19 (Fig. 6b). The mice with neurologic melioidosis were then evaluated using histological examinations, and bacterial loads were counted in the brain after another 24 h (n = 6, per group; protocols, see below).

### Cerebral cytokines, chemokines and CAMs

The mice were anesthetized with Zoletil (250 μg/g, intramuscular injection; Virbac Biotech. Inc., Taipei, Taiwan) and then transcardially perfused with 25 mL of PBS. Brain tissues were dissected, immediately homogenized, using a Pyrex brand Tenbroeck tissue grinder (Thermo Fisher Scientific Inc., Fremont, CA, USA), in the 1 mM phenylmethylsulfonyl fluoride (PMSF)-PBS solution and stored at −80 °C^40^. The levels of cerebral cytokines (TNF-α, IL-1α, IL-1β, IFN-γ, IL-12p70 and IL-17a), chemokines (MCP-1, MIG, MIP-1α, MIP-1β and RANTES) and CAMs (L-selectin, P-selectin, E-selectin, Intercellular Adhesion Molecule 1 [ICAM-1]) were determined at emission wavelengths of 423 and 578 nm using a Cytometric Bead Array kit (CBA; BD Biosciences; Franklin Lakes, NJ, USA) and flow cytometry (BD Biosystems FACSCalibur system, BD Biosciences) analysis^41^. The data were analyzed using an FCAP Array Software (version, 3.0; Bender Medsystems, Burlington, CA, USA), and standard curves were generated using the mixed cytokine/chemokine/CAM standard provided.

### Histological examination

The skulls of perfused animals were excised, fixed in 4% formaldehyde, and de-calcified with 10% trichloroacetic acid. The skull was then placed in a customized cutting box that allowed anatomically identical positions to be cut and embedded in wax. Paraffin sections were serially cut at a thickness of 1 μm per slice at the same position in each brain. Meningeal neutrophil infiltration was observed by standard hematoxylin and eosin staining. For immunohistochemistry (IHC), the sections were incubated with primary antibodies that were diluted in PBS according to the manufacturer’s instructions (Table S5). Colorimetric detection was performed using an UltraVision Quanto Detection System HRP Kit (Thermo Fisher Scientific Inc.) after the Quanto Substrate was added. The antibody/polymer conjugates were visualized by applying DAB Quanto Chromogen (Thermo Fisher Scientific) dissolved in PBS to the tissue sections for 20 min. The number of astrocytes (GFAP cells) in the hippocampus and microglia (Iba-1 cells) and endothelial (CD34) cells in the cerebral cortex were recorded. Images were captured from 10 randomly selected microscopic fields (200X) using a microscope (BX41; Olympus, Tokyo, Japan) equipped with a camera (TrueChromeII, Olympus). The averaged optical units from 10 fields (scan 3 squares, 200 μm^2^ per square, in each field) were determined using ImageJ software (https://imagej.nih.gov/ij/). The optical units were calculated using −log_10_(I_C_/I_0,C_) = A*c_C_ (I, transmitted light; IC, the intensity of the detected light after passing through the specimen; I_0,C_: the intensity of the light entering the specimen; A, the amount of stain with an absorption factor c; the subscript c indicates the detection channel)^40^. The maximum was set at 255 units in this study. Microglia were categorized as ramified, transitioning or resting. The proportion (%) of each category of microglia was calculated in 100 microglia per sample. The proportions determined in 6 independent tissues per group were averaged. To observe the morphology of astrogliosis or activated microglia, the positive controls were prepared from the mice with neurologic melioidosis that were induced *via* intravenous injection of *B. pseudomallei* for 10 d^10^.

### Vascular permeability

The endotoxemic mice were injected with Evan’s blue dye (2% in saline, 4 mL/kg) *via* the tail vein. After one hour, the mice were transcardially perfused with 25 mL of PBS, and the scalp and skull were carefully removed. The whole brain was photographed. Furthermore, the brain (ca. 0.4 g), heart (ca. 0.2 g), lungs (ca. 0.02 g), liver (0.5 g) and spleen (0.02 g) were homogenized by tissue grinders (Thermo Fisher Scientific Inc.) in 1.0 mL of 50% trichloroacetic acid and centrifuged at 12,000 g for 20 min. The supernatants were added to 0.3 mL of 100% ethanol, and the optical units at 630 nm were determined using a spectrophotometer (Spectra Max M5, Molecular devices, Sunnyvale, CA, USA).

### Brain-infiltrating leukocytes (BILs)

To collect BILs, the animals were perfused *via* the heart, and their brains were dissected and homogenized using the tissue grinders (Thermo Fisher Scientific Inc.)^40^. The leukocyte layers were harvested *via* centrifugation (500 × g for 30 min) in a 70/90% discontinuous Percoll-PBS solution, the myelin debris was removed using a mesh cell strainer filter (40 mm; Miltenyi Biotech., Bergisch Gladbach, Germany), and the samples were washed with Roswell Park Memorial Institute (RPMI) 1640 medium (Sigma, St. Louis, MO, USA). The cells were suspended in 1 mL of FACS (2% bovine serum albumin and 0.02% sodium azide in calcium and magnesium-free PBS) and then placed on 1 mL of Ficoll-Paque Plus solution (GE Healthcare Life Science Co., Chicago, IL, USA) for centrifugation (1,400 × g, 25 min). The BIL layer was washed with FACS and stored in a 2% FCS-PBS solution^42^. The number of BILs was counted by flow cytometry (Cell Lab Quanta SC; Beckman Coulter, Inc. Pasadena, CA, USA), and the results were analyzed using CXP software (Beckman Coulter, Inc.). *B. pseudomallei* is an intracellular pathogen that is capable of persisting in meningeal Ly6C monocytes, Ly6G neutrophils, F4/80 macrophages, CD3 lymphoid cells and CD19 B cells^11^ as well as in CD3^−^CD8^+^ NK cells^19^. Thus, the subpopulation of CD16/32^+^CD45^+^ cells, a population that included phagocytes, myeloid cells, NK cells, and some T cells and B cells, in BILs were stained with monoclonal PE (phycoerythrin)-conjugated CD45 and PE-Cy (Cyanine) 7-conjugated CD16/32 antibodies (Table S5) for 30 min on ice in the dark according to the manufacturer’s protocol. In particular, the high expression of CD45 on the surface of meningeal cells reflected the circulating leukocytes that infiltrated into the meninges^11^. Stained cells were evaluated by flow cytometry (Cell Lab Quanta), and the data were analyzed using CXP software (Beckman Coulter, Inc.)^11^.

### Bacterial loads in the brains

The mice were transcardially perfused and then sacrificed, and their brains were then excised. The bacterial load (CFU/g) in the brain (ca.0.4 g) was determined in each animal using sequential weighing *via* homogenization in 500 mL of PBS and a serial dilution protocol^11^. The limits of detection were 3 CFU/g.

### Adoptive transfer

*B. pseudomallei* can invade into progenitors or inflamed cells in bone marrow and then migrate to the BBB *via* a hematogenous route^11^. In preliminary tests, we found that over 95% of infected cells isolated from bone marrow of the melioidosis mice were survived after 12 h. However, if bone marrow Ly6C cells were infected with *B. pseudomallei in vitro* (MOI = 1:10, 1:1 and 10:1), those cells (>50%) would die within 6–8 h. Thus, the preparation of donor cells was chosen by *in vivo* but was not performed *ex vivo*. The Ly6C cells as the donors were obtained from the bone marrow of melioidosis mice, following previous preparation protocols^10^. Briefly, the mice (8-week-old females) were intravenously injected with 50 CFU of *B. pseudomallei* vgh19. On day 10 post-infection, nylon membranes (30 mm; Millipore, Billerica, MA, USA) were used to obtain bone marrow cells by filtering the liquid obtained from flushing. The cells were then treated with kanamycin (400 mg/ml for 2 h) to remove extracellular *B. pseudomallei*. Ly6C monocytes were isolated using an EasySep Mouse Positive Selection Kit (STEMCELL Tech. Inc., Vancouver, Canada) according to the kit’s instructions. Prior to adoptive transfer, the Ly6C cells were disrupted with 1%-Triton X, and the number of intracellular *B. pseudomallei* was determined by a serial dilution method^10,11^. In this study, we found that approximately 4.2 × 10^4^ cells contained 1 × 10^3^ CFU of *B. pseudomallei*. If the number of bacteria contained in the donor cells was over or under 15%, the data derived from adoptive transfer were not used in the analysis.

To evaluate the dose-response relationship, the 8-week-old female mice were adoptively transferred with 2.5 × 10^2^ (intracellular bacteria, ca. 30 CFU), 3.6 × 10^3^ (intracellular bacteria, ca. 300 CFU) or 4.2 × 10^4^ (intracellular bacteria, ca. 1000 CFU) donor cells or intravenously infected with free *B. pseudomallei* at 30, 300 or 1000 CFU from the tail vein (n = 6, per group). As controls, the mice were injected with PBS or empty Ly6C cells (2.5 × 10^2^–4.2 × 10^4^ cells). After 2 d, the mice were sacrificed, and the bacterial colonization in the brains was determined by the serial dilution method.

To evaluate the Trojan horse effect during endotoxemia, the donors (4.2 × 10^4^ cells) were adoptively transferred into recipient endotoxemic mice (n = 6, per group) *via* the tail vein. After 24 h, the mice were sacrificed. With respect to the bacterial loads, the bacterial colonization in the brain was determined by the serial dilution method. In cases of histological examination (protocols of sample preparation and H&E stains, see above), the meningeal structures in the whole brain were carefully observed for each mouse. Neutrophil infiltration appearing in the meninges anywhere in each sample (observed four sections, per sample) was counted as a positive result. The percentages (%) of meningeal neutrophil infiltration were calculated using 10 individuals examined per group in total^10^.

### Statistical analysis

All data are presented as the mean ± SD (standard deviation) derived from six tests performed in two independent experiments. The variable comparisons were analyzed using ANOVA and Tukey’s HSD test. One-to-one comparisons were performed using Student’s t-test. The difference in the presence or absence of meningeal neutrophil infiltration in two *Burkholderia* LPS-induced groups was examined by Fisher’s exact test (one-tailed). Significance was set at a level of p < 0.05.

## Electronic supplementary material

Supplemental information
