# Supplementary material for: Burkholderia pseudomallei-loaded cells act as a Trojan horse to invade the brain during endotoxemia
Source: Sci Rep. 2018 Sep 11;8:13632. doi: 10.1038/s41598-018-31778-8 (PMC6134107; doi:10.1038/s41598-018-31778-8)
Supplement: Supplementary file 1 — Supplemental information [file 41598_2018_31778_MOESM1_ESM.pdf]

***Burkholderia pseudomallei*-loaded cells act as a Trojan horse to invade the brain during endotoxemia**

Pei-Tan Hsueh[1,] Hsi-Hsun Lin[2,3], Chiu-Lin Liu[4], Wei-Fen Ni[4], Ya-Lei Chen[4]*, Yao-Shen Chen[1]*

Table S1. Cerebral cytokines, chemokines and cell adhesion molecules in endotoxemic mice

Brain tissue (24 h post-stimulation) induced by					
	B. pseudomallei **LPS (Mean±SD)**	***B. thailandensis*** **LPS (Mean±SD)**	***B. cenocepacia*** **LPS (Mean±SD)**	***B. multivorans*** **LPS (Mean±SD)**	***PBS***
**Cytokines (pg/g)**					
TNF-α	0.9±0.2[b]	0.8±0.1[b]	1.0±0.2[b]	2.2±1.0[a]	0.8±0.1[b]
IL-1α	23.4±2.1	19.3±4.8	15.7±5.0	15.5±4.0	10.3±7.1
IL-1β	21.1±1.6[b]	15.9±4.1[c]	22.7±2.1[b]	28.0±4.8[a]	8.3±1.2[d]
IL-12p70	14.8±3.0[b]	10.0±2.8[b]	10.9±1.8[b]	29.9±4.3[a]	12.3±1.8[b]
IL-17a	6.1±1.3	3.7±2.3	4.6±1.0	6.4±1.8	3.8±1.4
IFN-γ	2.1±0.8[bc]	1.9±0.4[bc]	2.8±0.9[b]	10.9±1.6[a]	1.1±0.1[c]
**Chemokines (pg/g)**					
MCP-1	229±61[bc]	212±29[c]	306±83[b]	1012±394[a]	118±40[d]
MIG	165±43[b]	145±63[b]	168±100[b]	289±57[a]	53±21[b]
MIP-α	48±20	39±11	27±3	39±9	14±5
MIP-β	13±5	9±2	7±2	11±5	5±2
RANTES	51±24[bc]	21±4[bc]	74±29[b]	237±64[a]	3±2[c]
**Cell adhesion molecules (pg/g)**					
L-selectin	7404±2952[b]	3642±363[c]	6902±824[bc]	14004±4567[a]	3631±936[c]
E-selectin	841±448	823±370	849±336	1009±344	118±48
P-selectin	468±50[b]	356±55[c]	284±41[d]	634±92[a]	62±12[e]
ICAM-1	752±64[c]	883±111[b]	375±86[d]	1066±114[a]	142±22[e]
**Brain tissue (48 h post-stimulation) induced by**					
	B. pseudomallei **LPS (Mean±SD)**	***B. thailandensis*** **LPS (Mean±SD)**	***B. cenocepacia*** **LPS (Mean±SD)**	***B. multivorans*** **LPS (Mean±SD)**	***PBS***
**Cytokines (pg/g)**					
TNF-α	1.0±0.2[c]	16.1±4.4[b]	1.1±0.2[c]	22.1±2.8[a]	0.8±0.1[d]
IL-1α	14.7±4.6[ab]	17.3±6.0[ab]	16.8±4.0[ab]	22.2±2.1[a]	11.0±4.0[b]
IL-1β	8.6±1.4[b]	9.6±3.1[b]	8.7±2.0[b]	25.7±4.1[a]	8.0±1.0[b]

IL-12p70	8.1±1.8[c]	8.7±1.0[bc]	9.3±1.9[bc]	37.9±3.6[a]	11.8±1.0[b]
IL-17a	7.3±1.7[a]	6.1±2.3[ab]	6.1±1.3[ab]	7.5±1.9[a]	4.5±1.5[b]
IFN-γ	1.2±0.3[c]	2.8±1.3[ab]	1.5±0.3[c]	2.9±0.8[a]	1.0±0.2[c]
**Chemokines (pg/g)**					
MCP-1	135±20[b]	174±38[b]	218±73[b]	1542±443[a]	151±37[b]
MIG	172±52[a]	178±30[a]	190±51[a]	209±46[a]	66±17[b]
MIP-α	44±7[ab]	49±11[a]	31±9[b]	57±16[a]	12±3[c]
MIP-β	7±2[b]	9±2[b]	7±3[b]	36±7[a]	5±1[b]
RANTES	20±5[bc]	45±7[b]	42±6[bc]	94±50[a]	3±2[c]
**Cell adhesion molecules (pg/g)**					
L-selectin	6501±4843[b]	3909±930[b]	3140±876[b]	15066±3603[a]	3217±741[b]
E-selectin	553±232[a]	551±247[a]	445±204[ab]	536±274[a]	138±35[b]
P-selectin	592±82[ab]	274±36[c]	285±59[c]	649±193[a]	65±16[d]
ICAM-1	541±168[c]	751±66[b]	403±116[c]	1064±107[a]	135±21[d]

The letters a-e represent significant differences at $p<0.05$ (ANOVA; Tukey's HSD test).

Table S2. Optical units of Evans blue in the homogenized organs of endotoxemic mice

	Optical units in endotoxemic mice induced by				
	***B. pseudomallei* LPS (Mean±SD)**	***B. thailandensis* LPS (Mean±SD)**	***B. cenocepacia* LPS (Mean±SD)**	***B. multivorans* LPS (Mean±SD)**	***PBS***
**Organs (weight/mL, dilution folds)**					
Liver (0.5 g/mL, 20X)	0.66±0.06[a]	0.67±0.10[a]	0.46±0.22[a]	0.86±0.32[a]	0.25±0.02[b]
Spleen (0.02 g/mL, 10X)	0.81±0.07[ab]	0.74±0.83[a]	0.46±0.16[bc]	0.76±0.10[a]	0.45±0.03[c]
Lung (0.02 g/mL, 10X)	0.80±0.15[ab]	0.77±0.10[ab]	0.64±0.21[ab]	0.86±0.20[a]	0.54±0.05[b]
Heart (0.2 g/mL, 2X)	1.01±0.06[a]	0.89±0.10[a]	0.46±0.16[b]	0.96±0.22[a]	0.52±0.06[bc]
Brain (0.4 g/mL, 1X)	0.59±0.05[b]	0.43±0.02[c]	0.44±0.05[c]	0.91±0.17[a]	0.22±0.02[d]

The letters a-e represent significant differences at $p<0.05$ (ANOVA; Tukey's HSD test).

Table S3. Summary of inflammatory expression in endotoxemic mice

Summary of expression of inflammatory indictors	
Cytokines	Orders of expression[a] Examined by ANOVA, Tukey's HSD
IL-1β	Bm > Bp = Bc > Bt > PBS
TNF-α, IL-12p70, IFN-γ	Bm > (Bp, Bt, Bc, PBS)
Chemokines	
MCP-1	Bm > (Bp, Bt, Bc) > PBS
RANTES	Bm > Bc ≥ (Bp, Bt, PBS)
MIG	Bm > (Bp, Bt, Bc, PBS)
CAMs	
P-selectin	Bm > Bp > Bt > Bc > PBS
ICAM-1	Bm > Bt > Bp > Bc > PBS
L-selectin	Bm > Bp > (Bc, Bt, PBS)
Glial cells	
GFAP signals	Bm > Bc > Bp > Bt > PBS
Iba1 transitioning cells	Bm > Bt > Bp = Bc > PBS
Endothelium	
CD34 signals	Bm > Bp ≥ (Bt, Bc, PBS)
Vascular permeability	
Optical units of Evan blue	Bm > Bp > Bc = Bt > PBS
Infiltrating cells	
Total BILs	Bm > Bp = Bt > Bc > PBS
$CD16/32^{+}CD45^{+}$ cells	Bm > Bp > Bt > Bc > PBS
Bacterial loads in the brains	
by infected cells	Bm > Bp > Bc > Bt
by free bacteria	Bm > Bp = Bt = Bc

	Examined by Fisher’s exact[b]
Meningeal neutrophil infiltration	
by infected cells	Bm(100%)^{ns}, Bp(100%)^{ns}, Bt(80%$^{)ns}$, Bc(80%)^{ns}
by free bacteria	Bm(50%)^{ns}, Bp(40%)^{ns}, Bt(40%)^{ns}, Bc(40%)^{ns}

a, Bm, Bp, Bc, Bt respectively represent as *B. multivorans*, *B. pseudomallei*, *B. cenocepacia*, *B. thailandensis* LPS-induced brains.

b, ns means no significance between any of paired *Burkholderia* LPS-induced brain.

Table S4. Comparison of the structures and in-vitro function of purified *Burkholderia* LPS

	Structures		In-vitro function
	Electrophoretic profiles[a] (ladders Ranged from)	Molar ratio[b]	[3-hydroxytetradecanoic acid][c] vs endotoxin units[d]
B. pseudomallei LPS	20 kDa - 40 kDa	1.42	R^2=0.99
B. thailandensis LPS	20 kDa - 40 kDa	1.30	R^2=0.98
B. cenocepacia LPS	20 kDa - 50 kDa	1.42	R^2=0.99
B. multivorans LPS	50 kDa - 100 kDa	1.25	R^2=0.99

a, on silver stain
b, [3-hydroxytetradecanoic acid] over [3-hydroxyhexadecanoic acid], concentrations determined by gas chromatography–mass spectrometry (GC-MS) analysis
c, determined by GC-MS analysis
d, determined using a Limulus coagulation enzyme kit (Seikagaku, Tokyo, Japan).

Detailed protocols, please refer to reference 14.

Table S5. Antibodies used in histological examinations and flow cytometry in this study

Target	Conjugated[a]	Isotype control	Clone	Species	Concentration	Representative	Company[b]
Ly6C,	PE	IgM κ	AL-21	Rat	2 μg/mL	Monocytes	BD Biosciences
CD45,	PE	IgG2a κ	MEC13.3	Rat	2 μg/mL	Leukocytes	BD Biosciences
CD16/32	PE-Cy	IgG2b κ	2.4G2	Rat	2 μg/mL	Fc receptors	BD Biosciences
CD34	None	IgG2a k	RAM34	Rat	2 μg/mL	Endothelium	BD Biosciences
GFAP,	None	IgG	Poly[c]	rabbit	2 μg/mL	Astrocytes	Abcam Co.
Iba1,	None	IgG2b	1022-5	rabbit	1 μg/mL	Microglia	Abcam Co.

a, PE: phycoerythrin; PE-Cy7: phycoerythrin-Cyanine 7

b, BD Pharmingen, Franklin Lakes, NJ, USA; Abcam Co., Cambridge, UK

c, polyclonal
